# Wnt5a mediates the effects of Bushen Huoxue decoction on the migration of bone marrow mesenchymal stem cells in vitro

**DOI:** 10.1186/s13020-018-0200-2

**Published:** 2018-08-29

**Authors:** Wei Shen, Hui Luo, Liangliang Xu, Zhifang Wu, Hongtai Chen, Yamei Liu, Lijuan Yu, Liuchao Hu, Bin Wang, Yiwen Luo

**Affiliations:** 1 Department of Traumatology, The Third Affiliated Hospital of Guangzhou University of Traditional Chinese Medicine, 22 Jiangnan-Xi Road, Guangzhou, 510240 Guangdong People’s Republic of China; 2 Key Laboratory of Orthopaedics & Traumatology, The First Affiliated Hospital of Guangzhou University of Chinese Medicine, Guangzhou, 510000 Guangdong People’s Republic of China; 3 Department of Diagnostics of Chinese Medicine, School of The Basic Medicine, Guangzhou, 510006 Guangdong People’s Republic of China

**Keywords:** Fracture, Bushen Huoxue decoction, Bone marrow mesenchymal stem cell, Migration, Wnt5a

## Abstract

**Background:**

Bushen Huoxue decoction (BHD) has a significant effect on fracture rehabilitation, yet its underlying mechanism is still unknown. The purpose of this study was to explore whether BHD promotes bone marrow mesenchymal stem cell (BMSC) migration through the Wnt5a signalling pathway.

**Methods:**

BHD was extracted by petroleum, and its composition was analysed. Cell viability in the presence of various concentrations of BHD for 24, 48 and 72 h was measured using a Cell Counting Kit-8 assay. Transwell assays and wound healing assays were used to observe the migration ability of BMSCs. Lentiviral vectors were used to knock down Wnt5a. Polymerase chain reaction and Western blot analyses were used to further compare Wnt5a signalling components at the mRNA and protein levels between groups.

**Results:**

BHD treatment groups showed increased migration ability and Wnt5a expression. Knocking down Wnt5a using a lentivirus significantly inhibited the effects of BHD, which implies that BHD promotes BMSC migration ability through activation of Wnt5a.

**Conclusions:**

BHD can enhance BMSC migration, possibly by activating Wnt5a signalling.

**Electronic supplementary material:**

The online version of this article (10.1186/s13020-018-0200-2) contains supplementary material, which is available to authorized users.

## Background

Bone fracture can severely impact a patient’s quality of life. Many patients suffer from fractures, which are both costly and require extensive time to heal. Bone fracture healing is a remarkably complex repair process which is similar to the embryonic development [1]. Delayed union or poor fracture healing poses a serious threat to the quality of life of patients. Bone fractures are repaired by two mechanisms: direct and indirect bone repair. Indirect fracture healing is initiated by an immediate inflammatory response, which results in the recruitment of bone marrow mesenchymal stem cells (BMSCs) to the injury site. Condensation is the pivotal stage in the development of skeletal and other mesenchymal tissues. It occurs when a previously dispersed population of cells gathers together to differentiate into a single cell/tissue type such as cartilage, bone, muscle [2]. BMSCs subsequently differentiate into chondrocytes, which produce cartilage and form a callus [3]. Bone formation begins when mesenchymal cells form condensations involving different cellular processes (migration, adhesion, proliferation, and growth) [2]. By identifying relevant cytokines and chemokines released from the bone injury region, BMSCs in niches can be activated and migrate to the injury region [4]. Therefore, intervening measures can accelerate BMSC migration processes and may contribute to bone repair.

Wnt signalling pathways can be divided into two categories: canonical (Wnt/β-catenin-dependent) pathways and non-canonical pathways. Non-canonical Wnt signalling pathways include the Wnt/PCP and Wnt/protein kinase C (PKC)-Ca^2+^ pathways [5]. Both pathways are linked to cell adhesion and movement [6, 7]. Wnt5a activates the latter pathways [6, 8, 9] and is closely associated with cell motility and invasion [10].

Bushen Huoxue decoction (BHD) has been used to treat craniocerebral diseases and ovarian diseases [11–13] and is helpful for treating bone diseases such as osteoarthritis and osteoporosis [14, 15]. BHD was first described by Zhuquan Zhao in the Qing Dynasty. Since then, it has been widely used in patients with fractures, especially elderly patients. According to traditional Chinese medicine, BHD can promote blood circulation, help alleviate swelling at the fracture site and accelerate bone repair. Studies demonstrated that BHD treated group could obviously promote differentiation, proliferation and mineralization of osteoblasts through activation of Wnt/β-catenin signaling pathway and can also improve cartilage metabolism in experimental rabbits and possesses osteo-chondric protective effects in antagonizing peroxidation injury [16, 17]. In our previous study, we compared the migration ability of BMSCs treated with BHD extracted with four different solvents (petroleum ether, ethyl acetate, absolute alcohol and water). We found that BHD extracted with petroleum induced the greatest improvement in cell migration in a dose-dependent manner [18]. However, the underlying mechanism is still unknown. The purpose of this study was to explore this unknown mechanism. We hypothesized that BHD promotes cell migration ability by activating Wnt5a.

## Methods

The minimum standards of reporting checklist contains details of the experimental design, and statistics, and resources used in this study (Additional file 1).

### BHD preparation and detection of herbal ingredients

Eleven Chinese herbs (Radix Rehmanniae 18 g, Semen Cuscutae 18 g, Fructus Psoraleae 18 g, Eucommia ulmoides 6 g, Fructus Corni 6 g, Herba Cistanches 6 g, Fructus Lycii 6 g, Radix Angelicae Pubescentis 6 g, Radix Angelicae Sinensis 6 g, Myrrha 6 g, and Flos Carthami 3 g) were purchased from The Third Affiliated Hospital of Guangzhou University of Chinese Medicine. After drying for 24 h and being pulverized into powder, the total herbs were wrapped with filter paper and transferred to a Soxhlet apparatus. The components were extracted using the Soxhlet extraction method in petroleum [19]. The extract was concentrated by rotary evaporation and plaster precipitation, and the product was weighed. One gram was dissolved in petroleum and analysed by gas chromatography–mass spectrometry (GC–MS). The remaining product was dissolved in dimethylsulfoxide and filtered through a 0.22-μm syringe filter (Millex-GP, USA) before use. The concentration of the storage solution was 400 μmol/ml and was diluted to the needed concentration before use.

### BMSC isolation and culture

This study was approved by the Animal Care and Use Committee of Guangzhou University of Traditional Chinese Medicine. BMSCs were isolated from Sprague–Dawley rats (male, 4 weeks old, 60–80 g) in a sterile environment (n = 30). Rats were euthanized by carbon dioxide inhalation. The bone marrow of the bilateral femoral and tibial shafts was flushed out with serum-free low-glucose Dulbecco’s modified Eagle’s medium (DMEM) to obtain a single-cell suspension. The obtained bone marrow solution was centrifuged at 1200 rpm for 6 min, the supernatant liquid was removed, and the cell pellets were resuspended in glucose-DMEM supplemented with 10% foetal bovine serum, 1% β-mercaptoethanol and 1% penicillin and streptomycin (all from Gibco, USA). The medium was discarded after 24 h of primary culture and then changed once every 3 days. On day 4, the non-adherent cells were washed out with phosphate-buffered saline (PBS), and the adherent cells were further expanded until reaching 80% confluence. Cells were digested with a 0.25% trypsin solution and observed under an inverted microscope. The digestion was terminated by addition of low-glucose DMEM containing 10% serum when cells became round and detached. A single-cell suspension was prepared and subcultured at a ratio of 1:2. The medium was changed several times to obtain pure BMSCs. Cells from passages 3 to 5 were used in the study.

### Osteoblast and adipocyte differentiation

BMSCs were cultured and seeded in 6-well plates in l-DMEM containing 10% FBS and 1% penicillin–streptomycin for 24 h. The medium was replaced by osteoblast media or adipocyte media when cells became confluent. The osteoblast media contained 10^−8^ M dexamethasone, 10 mM β-glycerophosphate and 0.05 mM l-ascorbic acid (all from Sigma-Aldrich, MO, USA), while the adipocyte media contained 10^−6^ M dexamethasone, 0.5 mM isobutylmethylxanthine, 100 μM indomethacin and 10 mg/l insulin (all from Sigma-Aldrich, MO, USA). The medium was changed every 3 days. On the 21st day, alizarin red and oil red O staining were performed to observe the differentiation of BMSCs.

### Characterization of BMSCs

Cells were harvested by trypsin, resuspended in cold PBS, and then incubated with the corresponding FITC/PE-conjugated antibodies for the stem cell markers CD-90, CD-44, the endothelial marker CD-34, and the haematopoietic marker CD-45. Cells were analysed using a BD FACSCanto flow cytometer.

### Detection of cell proliferation

BMSCs were inoculated at 2 × 10^5^/ml in 96-well culture plates (approximately 2000 cells in 100 μl of medium per well). Then, 100 μl of serum-free medium was added, and cells were starved for 12 h. The medium was replaced with complete medium containing different concentrations of BHD (0, 1, 10, 25, 50, 100 and 150 μg/ml). Ten microlitres of CCK8 dye was add to each well after 24, 48 h, and cells were cultured for an additional 3 h. The absorbance was recorded at a wavelength of 450 nm using a microplate reader to determine the level of proliferation in each well (n = 6).

### Scratch wound healing assay

BMSCs were seeded in 6-well plates and cultured until 95% confluence. The medium was displaced by serum-free l-DMEM for 12 h. A scratch wound was created with a micropipette tip. The cells were treated with different concentrations of BHD (0, 25, 50, 100, and 150 µg/ml), and the scratch area was observed under a phase contrast microscope and photographed (n = 6).

### Cell migration assay

Cell migration ability was evaluated using in Transwell plates with a pore size of 8 μm (Corning Costar, Cambridge). Cells were digested and resuspended at 1 × 10^5^/ml in serum-free medium in the upper chamber. The upper chamber was then loaded with 8 × 10^4^ BMSCs in 200 μl serum-free medium. After culturing in an incubator for 2 h, the lower chamber was loaded with 700 μl complete medium containing different concentrations of BHD. The plates were incubated at 37 °C in 5% CO_2_ for 10 h. The upper surface of the membrane was then gently scraped using a cotton swab to remove the non-migrated cells and washed with PBS. The membrane was then fixed in 4% paraformaldehyde for 30 min, followed by staining with Giemsa stain. Migrated cells were observed and photographed under a phase contrast microscope. The number of migrated cells was determined by averaging five random fields per well (n = 6).

### Lentivirus transfection

To obtain BMSCs with silenced Wnt5a expression, BMSCs were cultured and transfected with Wnt5a-specific short hairpin RNA (sh-Wnt5a) lentiviral vectors. Green fluorescent protein (GFP) was expressed in the lentiviruses and was used to evaluate BMSC transduction efficiency. The transfection efficiency was tested by quantitative RT-PCR. Transfections were performed in BMSCs with a multiplicity of infection (MOI) of 6:1, following the manufacturer’s instructions. Lentiviral vectors were purchased from GeneChem (Shanghai, China). The medium containing lentiviral vectors was replaced with complete medium after 12 h.

### Real-time PCR analysis

Total RNA was extracted from BMSCs using TRIzol reagent (Invitrogen, Carlsbad, CA, USA) according to the manufacturer’s instructions. The RNA concentration was measured using an ultraviolet spectrophotometer (BioSpec-nano). cDNA was produced from the total RNA using PrimeScript RT Master Mix (TaKaRa, Tokyo, Japan). Primer sequences are shown in Table 1. The expression levels of Wnt5a, PKC, JNK and CaMKII were analysed. The ∆∆Ct method was used to calculate the expression of mRNAs relative to GAPDH. All steps were performed under RNase-free conditions.

**Table 1:** List of primer sequences for RT-PCR

Primer name	Sequence	Product length (bp)
WNT5A forward	5′-CGAAGACGGGCATCAAAGA-3′	97
WNT5A reverse	5′-TGCATCACCCTGCCAAAGA-3′	
JNK forward	5′-GGAGCGAACTAAGAATGGCG-3′	106
JNK reverse	5′-CATGTCATTGACAGACGGCG-3′	
CaMKII forward	5′-ATGGATGGAAATGGAATGCC-3′	102
CaMKII reverse	5′-CCCCGAACGATGAAAGTGAA-3′	
PKC forward	5′-AAGGTGGTCCACGAGGTGAA-3′	100
PKC reverse	5′-TTCCAATGCCCCAGATGAAG-3′	
GAPDH forward	5′-AGGGCTGCCTTCTCTTGTGA-3′	110
GAPDH reverse	5′-AACTTGCCGTGGGTAGAGTCA-3′	

### Western blotting

For Western blotting, we used RIPA buffer (Boston BioProducts, Ashland, MA, USA) to extract the total protein of BMSCs. The protein concentration was measured by a BCA Protein Assay kit (Pierce, Rockford, IL, USA). Equivalent masses of protein samples (30 μg) were separated by 10% SDS-PAGE and electrophoretically transferred onto PVDF membranes (Millipore, Billerica, MA, USA). The PVDF membranes were blocked in TBST containing 5% skim milk for 1 h and then incubated with mouse anti-Wnt5a (1:1000, Abcam), rabbit anti-SAPK/JNK (1:1000, CST), rabbit anti-CaMKII (1:1000 CST), mouse anti-PKC (1:1000, Millipore) and rabbit anti-GAPDH (1:4000, Abcam) primary antibodies overnight at 4 °C. The membranes were washed in TBST and incubated with a corresponding secondary antibody for 1 h at room temperature. Bands were visualized using an enhanced chemiluminescence kit according to the manufacturer’s protocol.

### Statistical analysis

Data are presented as the mean ± SD. One-way ANOVA was used for multiple comparisons. A value of P < 0.05 was considered statistically significant.

## Results

### Analysis of the herbal formula: BHD chemical ingredients

**Fig. 1:**
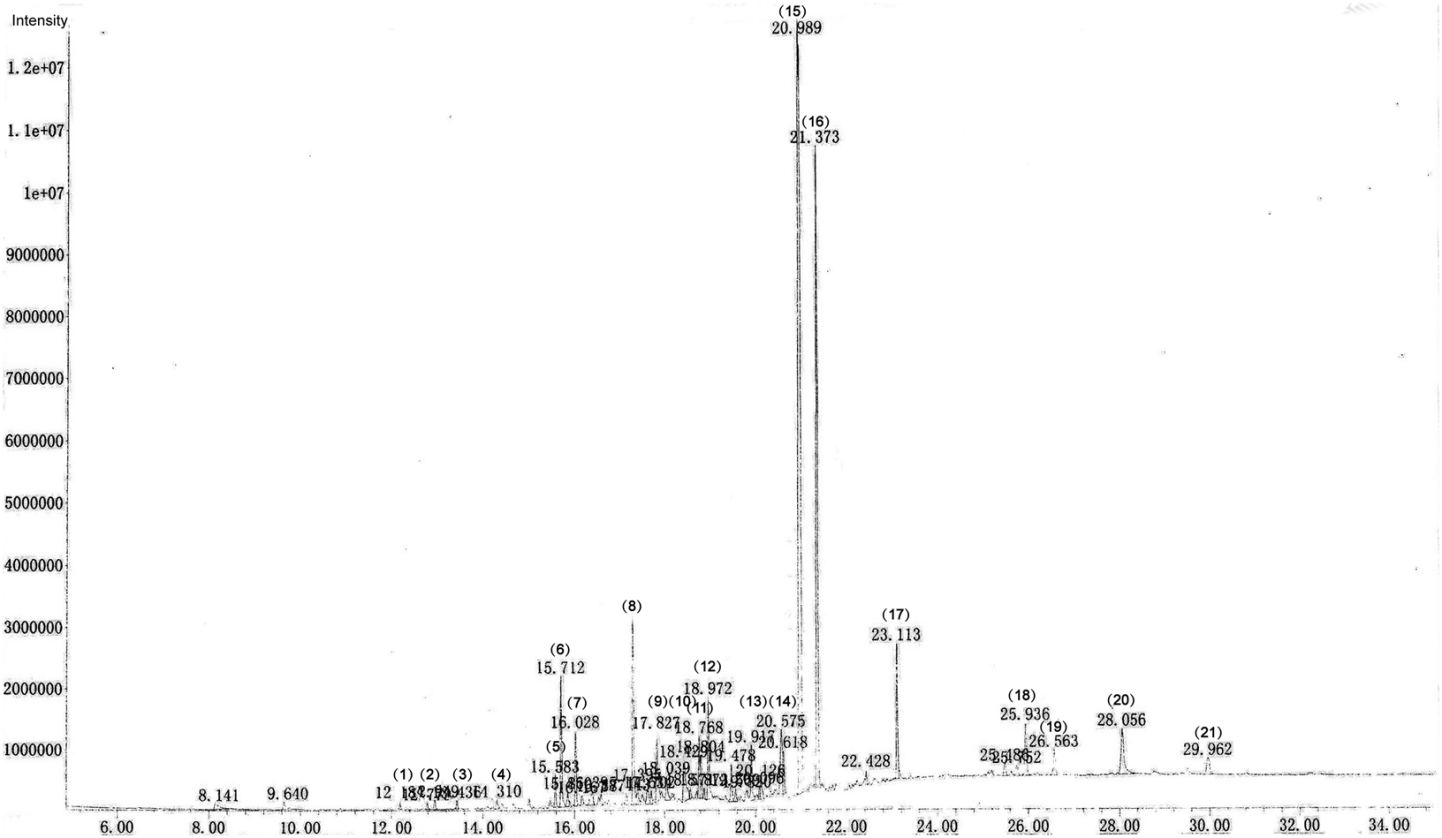
The GC–MS fingerprint shows the active ingredients of BHD

BHD extracted with petroleum ether was sent to the Chinese National Analytical Center, Guangzhou, and was analysed by GC–MS. A total of 45 peaks were shown in GC/MS spectrum. Standard analytical procedures were used for screening of ingredients. The spectrum of components was compared with spectrum stored in the NIST library version. The eluted compounds were characterized on the basis of their molecular formula, structure, retention time, and peak % area. A total of 21 distinct peaks are reflected. Twelve constituents were found in the fingerprint (Fig. 1) and are summarized in Table 2.

**Table 2:** Chemical components of BHD

No.	Identification	Peak % area
1.	δ-Elemene	0.19
2.	α-Copaene	0.16
3.	Caryophyllene	0.19
4.	β-selinene	0.29
5.	Caryophyllene oxide	0.74
6.	Curzerenone	3.21
7.	Unknown ingredient	1.72
8.	3-Butylphthalide; 3-butyl-1(3*H*)-isobenzofuranone	5.22
9.	Isopsoralen	2.43
10.	Psoralen	2.32
11.	Unknown ingredient	1.25
12.	Unknown ingredient	2.57
13.	Unknown ingredient	1.59
14.	Unknown ingredient	2.63
15.	Bakuchiol	34.92
16.	Osthole	18.54
17.	Unknown ingredient	3.28
18.	Decursin	1.87
19.	Unknown ingredient	0.99
20.	Unknown ingredient	2.73
21.	Unknown ingredient	0.95

### Identification of BMSCs

**Fig. 2:**
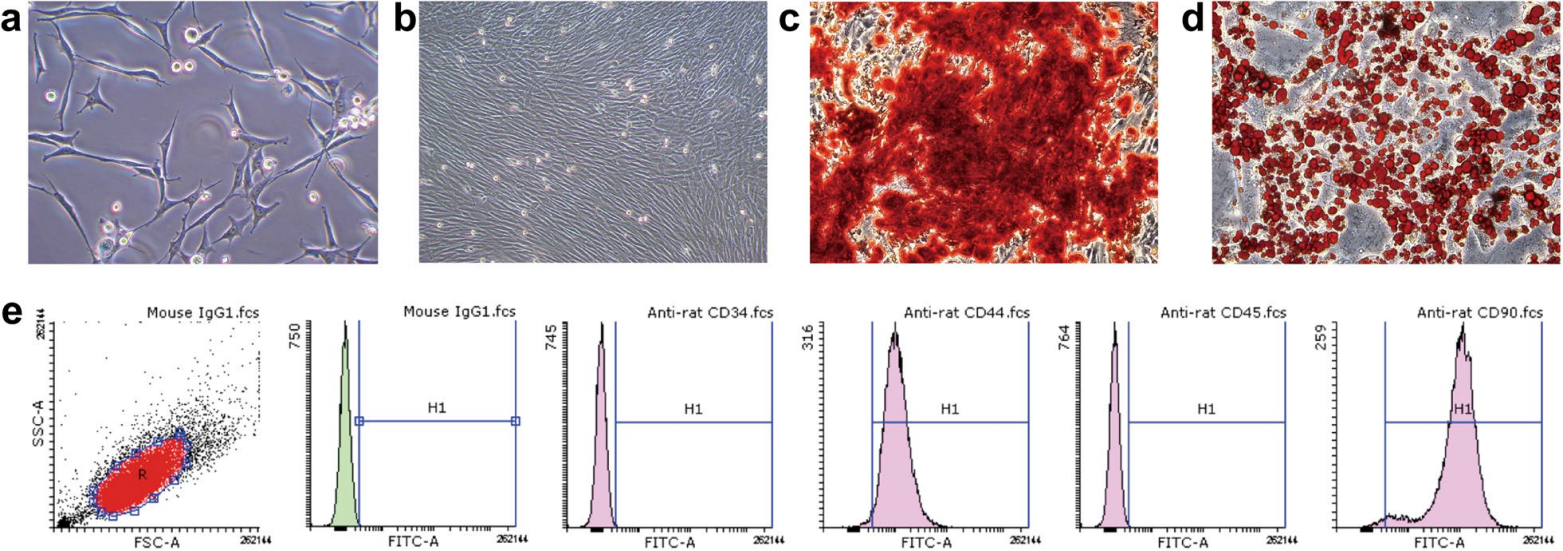
**a** BMSCs exhibited a fusiform cell morphology. **b** BMSCs at passage 3 and 95% confluence. **c** Alizarin red staining shows that the cells were capable of osteogenesis. **d** Oil red O staining shows that cells were also adipogenic. **e** Cells were positive for CD-44, and CD-90 and negative for CD-34 and CD-45

BMSCs were cultured and passaged until passage 3. They showed a fusiform cell morphology (Fig. 2a, b), and cells had both osteogenic (Fig. 2c) and adipogenic differentiation ability (Fig. 2d). BMSC surface markers were analysed by flow cytometry. The cells were positive for CD90 (98.63%) and CD44 (98.48%) but negative for CD34 (0.25%) and CD45 (0.25%) (Fig. 2e), which morphologically and immunophenotypically demonstrated that the cells were BMSCs.

### Treatment of BMSCs with different concentrations of BHD

**Fig. 3:**
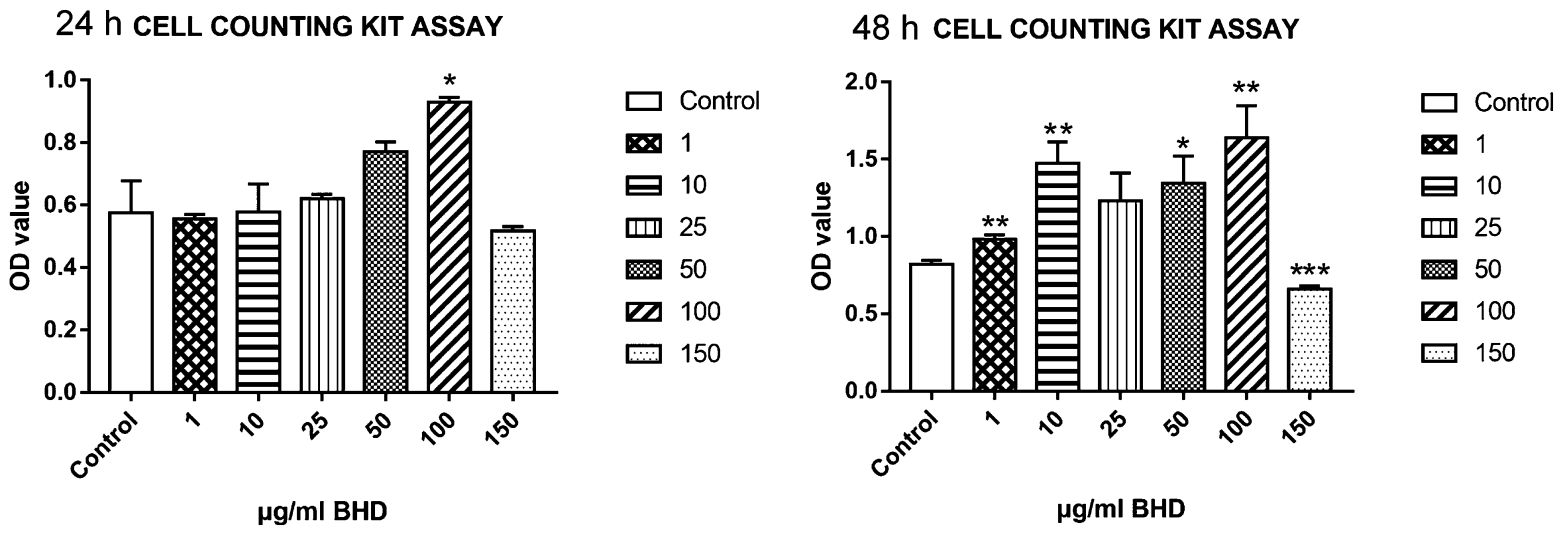
A Cell Counting Kit-8 was used to detect cell viability. Exposure to high-dose BHD for 24 h and 48 h inhibited cell viability. Treatment with 100 µg/ml BHD increased cell viability at 24 h and 48 h. *P < 0.05, **P < 0.01, ***P < 0.0001 for the treatment groups vs. the control group (n = 6)

As shown in Fig. 3, cell viability peaked when BMSCs were treated with 100 μg/ml BHD for 24 h and 48 h (Fig. 3). These results show that low-dose BHD is not toxic to cells and can promote cell proliferation within 48 h of treatment.

### Silencing Wnt5a inhibits BMSC migration

**Fig. 4:**
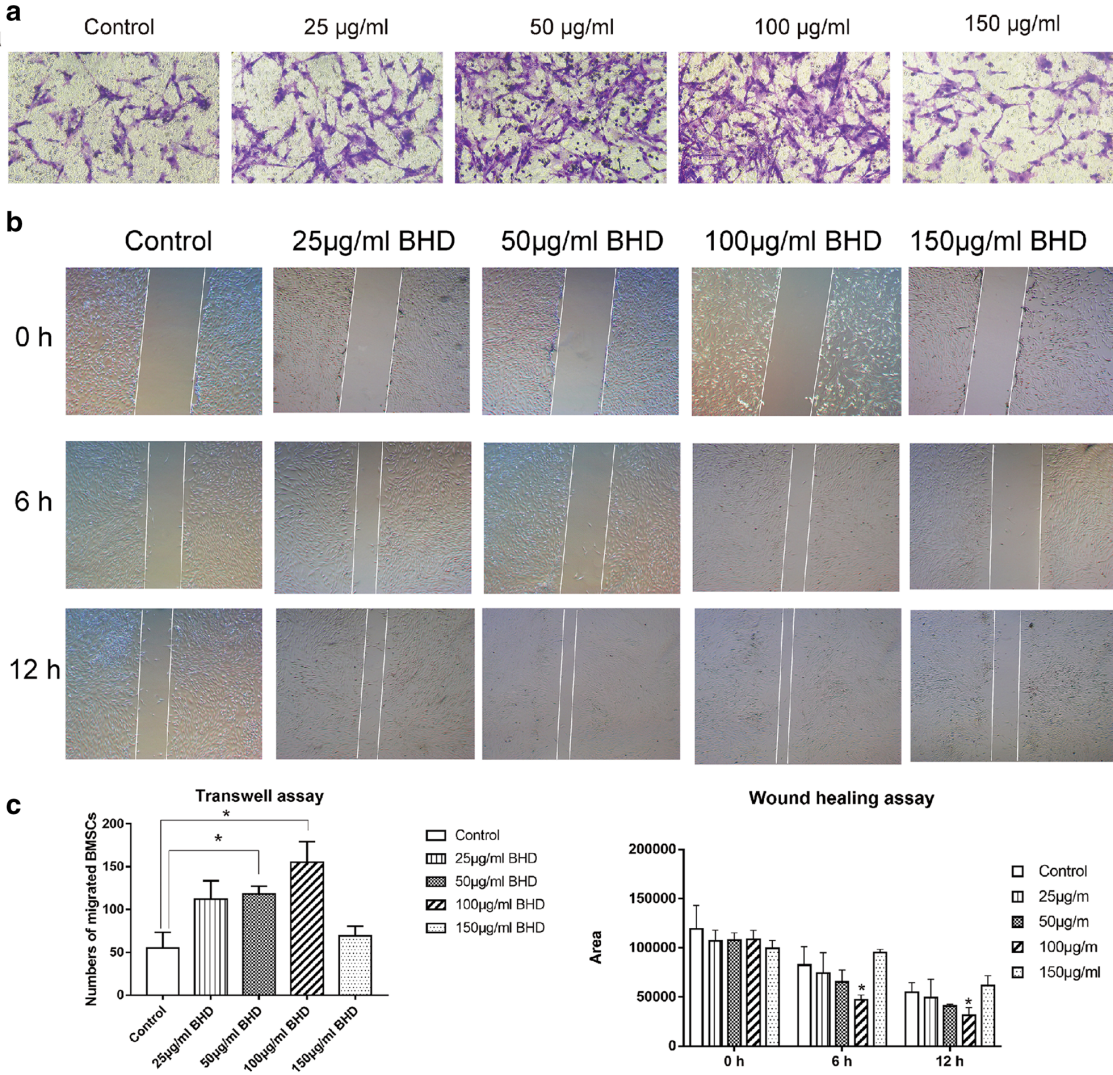
BHD-treated groups showed a stronger migration ability, especially the 100 µg/ml BHD group. **a** Transwell assays showed that BHD enhanced cell migration ability. **b** Dynamic migration in the scratch wound healing assay. BMSCs treated with 100 µg/ml BHD showed outstanding performance with regard to healing speed. **c** The data for transwell assay and wound healing assay. *P < 0.05 for the treatment groups vs. the control group

**Fig. 5:**
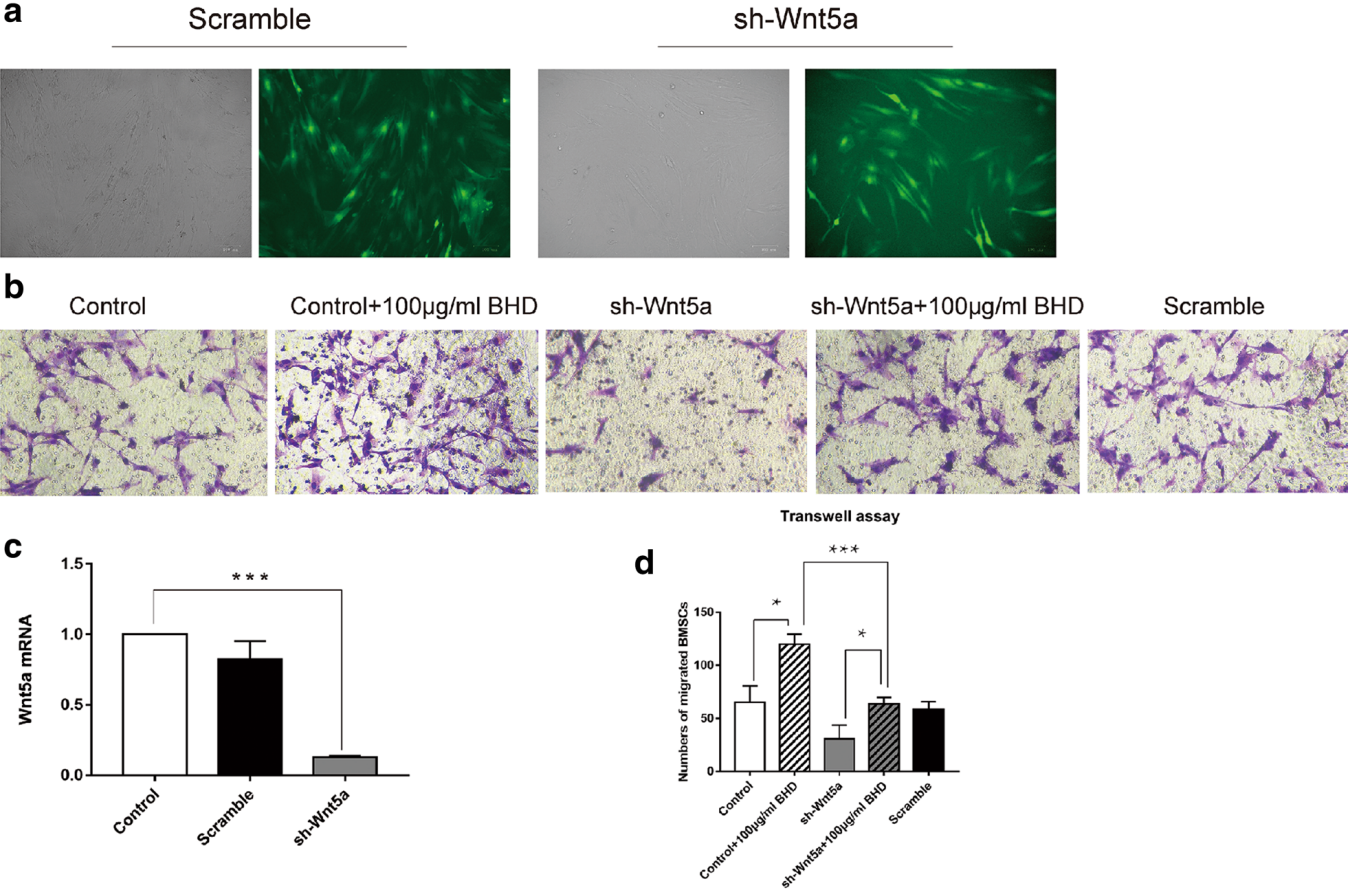
Silencing Wnt5a inhibited BMSC migration. **a** Green fluorescent protein was used to demonstrate transfection efficiency. The cells were successfully transfected by Wnt5a-specific short hairpin RNA (sh-Wnt5a) lentiviral vectors. **b** Cell migration ability was promoted by 100 µg/ml BHD in both the control and sh-Wnt5a groups. Silencing Wnt5a reduced cell migration ability. **c** The efficiency of shRNA activity was analysed by real-time PCR. The expression of Wnt5a was significantly reduced. **d** The data for transwell assay *P < 0.05, ***P < 0.0001

Different concentrations of BHD (150, 100, 50, 25 μg/ml) were added to the lower chamber of the Transwell plate, and migrated cells were counted after staining and photographing. Treated groups showed a significant acceleration of BMSC migration compared with that in the control group, especially in the 100 μg/ml BHD group (Fig. 4a). The scratch wound healing assay showed comparable results (Fig. 4b). To observe whether BHD could activate Wnt5a expression, BMSCs were transduced with sh-Wnt5a. After transfection by lentiviral vectors (Fig. 5a), sh-Wnt5a and sh-scrambled BMSCs were harvested and compared regarding Wnt5a expression. sh-Wnt5a BMSCs exhibited significantly reduced Wnt5a expression (Fig. 5c). Then, a Transwell assay was performed to compare their migration ability with the 100 μg/ml BHD group (Fig. 5b), and the data showed that silencing Wnt5a significantly inhibited the migration of BMSCs (Fig. 5d).

### BHD activates Wnt5a-related gene expression

**Fig. 6:**
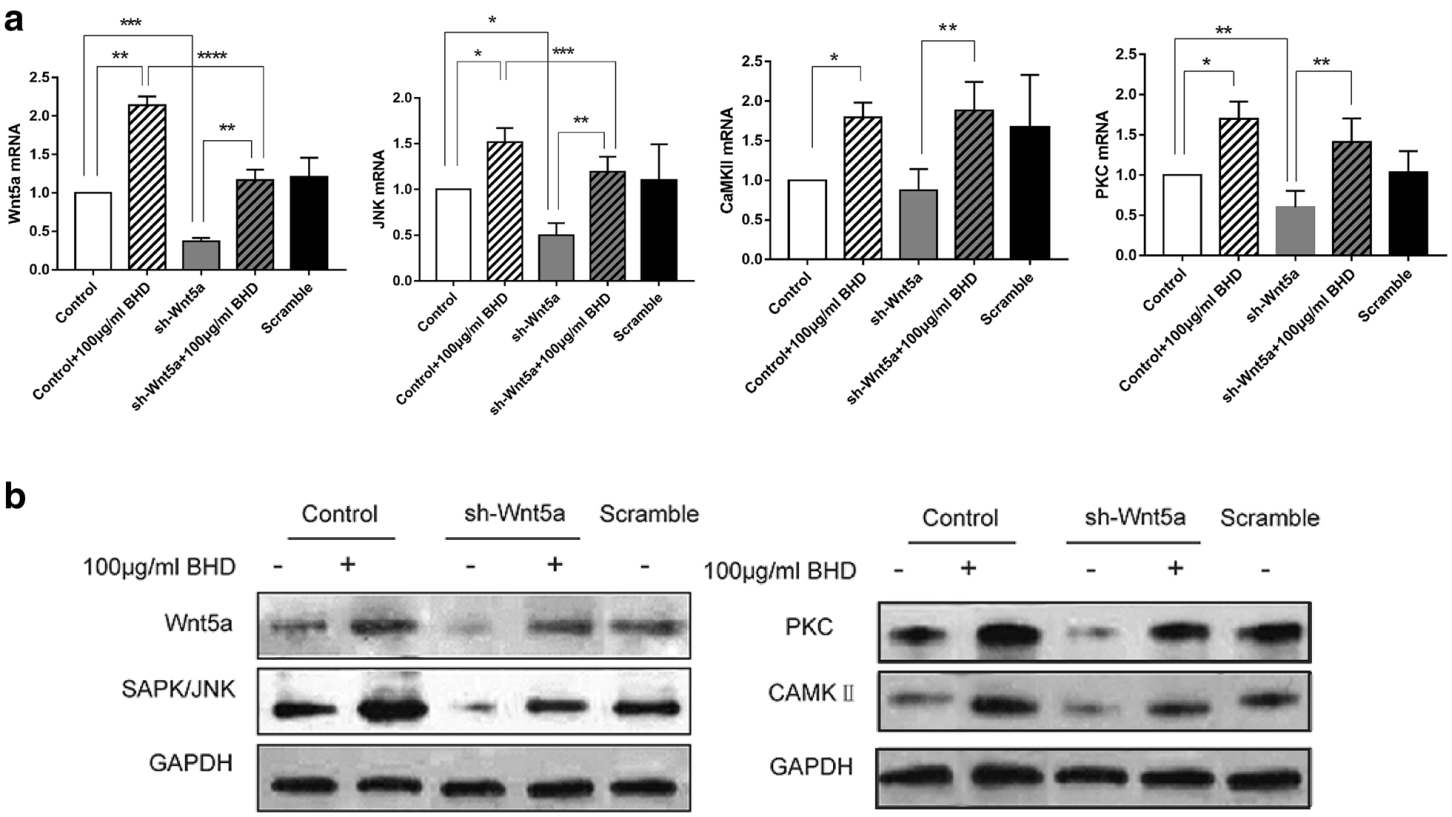
BHD activates Wnt5a and its associated pathway. **a** mRNA expression of Wnt5a, JNK, PKC and CaMKII. *P < 0.05, **P < 0.01, ***P < 0.0001. **b** Expression levels of Wnt5a, SAPK/JNK, PKC and CaMKII in all groups. The data show that BHD upregulated Wnt5a, SAPK/JNK, PKC and CaMKII

To determine whether BHD can active Wnt5a, we treated BMSCs with 100 μg/ml BHD. Wnt5a, SAPK/JNK and CaMKII expression were all significantly increased at the mRNA level (Fig. 6a).

### Western blot analysis

Comparable results at the protein level were observed in Western blot analyses of Wnt5a, SAPK/JNK, CaMKII and PKC. The data showed a parallel result to the mRNA data, which indicates that BHD can act on Wnt5a and upregulate its signalling proteins, SAPK/JNK, CaMKII and PKC (Fig. 6b).

## Discussion

BHD has been used to promote fracture healing by doctors of traditional Chinese medicine since it was described by Zhuquan Zhao in the Qing Dynasty. Because of its curative effect on fracture healing, it currently remains widely used in China as a complementary treatment for fractures. Fracture patients who receive conservative treatment in China are more likely to use BHD. Many researchers in China have observed its effect in patients with various types of fractures. Despite its long history of successful use, the underlying mechanism by which BHD promotes fracture healing is still unknown. In this study, we hypothesized that BHD enhances BMSC migration through activating the Wnt5a signalling pathway to facilitate bone healing. BHD contains 11 herbs, and after decoction, additional active ingredients were obtained. The main components of BHD are shown in Table 2. Several studies have shown a correlation between BHD components and bone diseases, mainly regarding fracture healing. Bakuchiol, the main ingredient of BHD, was shown to exhibit oestrogenic activity in both in vivo and in vitro models [20, 21]. Sunyer et al. found that oestrogen exerted bone-protective effects [22], which may contribute to fracture repair. Bakuchiol also exhibits anti-microbial, anti-inflammatory, anti-oxidative, anti-osteoporosis, and anti-depression or anti-stress activities [23]. Osthole has been widely studied in bone diseases because it may activate the β-catenin–BMP-2 signalling pathway to regulate osteoblast differentiation in vitro [24]. Zhang et al. demonstrated that osthole promotes the progression of repair by enhancing intracartilaginous ossification [25].

Many studies on the involvement of the Wnt pathway in the process of bone repair have been reported [26], and most are associated with the canonical Wnt/β-cat pathway. However, few studies have found a role for a non-canonical Wnt pathway, such as that of Wnt5a, in regulating the migration of BMSCs. The Wnt5a pathways are classified into nine categories, e.g., Wnt5a/planar cell polarity (PCP) signalling, Wnt5a/Ca^2+^ signalling, and Wnt5a/atypical PKC signalling [27]. The Wnt/PCP pathway has been linked to Wnt5a in vertebrates and can regulate cell migration and intercalation [6]. The Wnt/PCP pathway starts a signalling cascade that eventually leads to JNK activating the final transcription factor c-JUN (AP1) [5, 6]. Activation of the Wnt5a pathway can induce the release of calcium from intracellular stores and the upregulation of target proteins (PKC). In this study, we found that BHD-treated BMSCs showed better migration in the control group than in the sh-Wnt5a group. The expression of Wnt5a, PKC, CaMKII, and SAPK/JNK showed similar trends at the mRNA and protein levels, which indicates that BHD activates the Wnt5a/PCP and Wnt5a/PKC-Ca^2+^ pathways to enhance BMSC migration capacity.

## Conclusion

Bone fracture healing is a unique physiologic process which the migration of BMSCs is essential during the inflammatory and hematoma stages. From ancient times to the present, herbs have played an important role in the treatment of diseases in China and have achieved remarkable results. With the development of modern medicine, especially surgical techniques, Chinese herbal medicine treatments can often play a supplementary role in clinical treatment. For example, BHD aids in healing bone fractures. In the present study, we investigated the effect of BHD on BMSC migration and found that BHD promoted BMSC migration through activation of the Wnt5a signalling pathway. Further research is needed to determine which herbal ingredient is responsible for this improvement in the curative effect.

## Additional file

**Additional file 1.** Minimum standards of reporting checklist.

## Abbreviations

BHD: Bushen Huoxue decoction
BMSCs: bone marrow mesenchymal stem cells
Wnt5a: Wnt family member 5a
PCP: planar cell polarity
JNK: c-Jun N-terminal kinase
CaMKII: calcium–calmodulin-dependent protein kinase II
PKC: protein kinase C
GC–MS: gas chromatography–mass spectrometry
DMEM: Dulbecco’s modified Eagle’s medium
BCA: bicinchoninic acid
PBS: phosphate-buffered saline
TBST: tris-buffered saline plus Tween 20
SDS-PAGE: sodium dodecylsulfate–polyacrylamide gel electrophoresis
GFP: green fluorescent protein
